# Efficacy of *Pediococcus acidilactici* PA53 in preventing high-fat diet-induced non-alcoholic fatty liver disease in mice

**DOI:** 10.3389/fimmu.2025.1743709

**Published:** 2026-01-12

**Authors:** Yue Niu, Peng Liu, Yu Chen, Yichen Yao, Shurui Bu

**Affiliations:** 1 Department of Gastroenterology, Jinshan Hospital, Fudan University, Shanghai, China; 2 Department of General Practice, Jinshan Hospital, Fudan University, Shanghai, China; 3 Department of Traditional Chinese Medicine, Jinshan Hospital, Fudan University, Shanghai, China

**Keywords:** early prevention, gut microbiota, NAFLD, *Pediococcus acidilactici* PA53, probiotics

## Abstract

**Aim:**

To investigate the therapeutic and preventive effects of the probiotic *Pediococcus acidilactici* PA53 on high-fat diet (HFD)-induced non-alcoholic fatty liver disease (NAFLD) in mice, and to evaluate its efficacy compared to curcumin (a well-recognized anti-NAFLD agent), thereby advancing the mechanistic and practical understanding of probiotic-based interventions for metabolic liver diseases.

**Methods:**

The experiment comprised five groups: Control group, Model group, PA53 prevention group, PA53 treatment group, and curcumin group. NAFLD was induced by feeding mice a high-fat diet (HFD), with body weight recorded weekly. At the end of the experiment, serum, liver, and ileum samples were collected from the mice. The levels of serum inflammatory factors, including tumor necrosis factor-α (TNF-α), interleukin-1β (IL-1β), interleukin-6 (IL-6), and interferon-inducible protein-10 (IP-10), as well as related blood biochemical markers such as alanine aminotransferase (ALT), glucose (GLU), triglyceride (TG), cholesterol (CHO), and low-density lipoprotein cholesterol (LDL-C), were measured. Hepatic lipid deposition was evaluated using hematoxylin and eosin (H&E) staining and oil red O staining. Additionally, fecal samples were collected at multiple time points and subjected to 16sRNA sequencing to assess changes in the gut microbiota.

**Results:**

PA53 significantly attenuated body weight gain, liver lipid accumulation, serum inflammatory cytokine levels while normalizing gut microbiota composition in mice, bringing these parameters closer to those of the control group. Compared to curcumin, PA53 proved more effective in controlling body weight gain and reducing liver fat accumulation. Furthermore, the preventive regimen yielded better outcomes than the therapeutic effect. While PA53 was less effective than curcumin in modulating certain immune responses and reducing specific cytokines (such as IL-6, IL-1β, TNF-α and IP-10), it still exerted significant anti-inflammation effects.

**Conclusion:**

In conclusion, PA53 demonstrates potential efficacy in mitigating HFD-induced NAFLD in mice, with its preventive effect appearing comparatively more prominent. Notably, PA53 exhibited superior performance over curcumin in key metabolic endpoints, which furnishes novel empirical evidence for its putative role in NAFLD management and augments the current body of knowledge regarding probiotic-based therapeutic strategies for metabolic liver diseases.

## Introduction

Metabolic dysfunction-associated steatotic liver disease (MASLD) and Metabolic dysfunction-associated fatty liver disease (MAFLD) are the updated terminologies for the condition previously referred to as non-alcoholic fatty liver disease (NAFLD) (1). To ensure consistency with the extensive body of prior literature in the field and maintain clarity for readers familiar with established works, the term NAFLD is retained throughout this manuscript, with full acknowledgment of its updated nomenclature. NAFLD refers to the accumulation of fat in the liver that is not due to alcohol consumption and is one of the most common liver diseases worldwide (2). The prevalence is particularly high in individuals with metabolic disorders such as obesity, type 2 diabetes, dyslipidemia and hypertension (3). Worldwide, the estimated prevalence of NAFLD is around 25% (4). Early stages of the disease are characterized by simple fatty liver, which can develop into non-alcoholic steatohepatitis (NASH) as the disease progresses and can eventually lead to cirrhosis and liver cancer (5). Ezdiffra is the first drug to be approved by the FDA for the treatment of NASH (6). This development is encouraging, as it is intended for the treatment of adult patients with non-cirrhotic NASH and moderate to advanced liver fibrosis (corresponding to stages F2 to F3). However, the most important measures for the treatment and prevention of NAFLD remain dietary changes and regular exercise (7). Currently, there is no uniform clinical standard for the optimal dietary intervention strategy for NAFLD (8). Even if we were to establish a standard dietary strategy, developing and implementing personalized dietary plans for the general population without medical training would be challenging. Therefore, to improve public health and reduce the incidence of liver cirrhosis and even liver cancer, accelerating research on comprehensive early clinical prevention measures for NAFLD is crucial.

The liver and intestine are connected via the portal vein and bile duct system, establishing intricate and complex interactions between these two organs (9). A substantial body of research suggests that dysbiosis of the gut microbiota is closely associated with the onset and progression of NAFLD (10, 11). Various drugs or therapeutic approaches can also treat NAFLD by improving the composition of the gut microbiota (12–14). Gut microbiota dysbiosis can modulating intestinal permeability, thereby increasing the liver’s exposure to harmful substances. It can also alter the expression levels of certain gut metabolites such as short-chain fatty acids, alcohol and choline, leading to intestinal inflammation or other immune dysfunctions. These factors contribute to liver damage, resulting in liver inflammation and lipid accumulation (15).

Based on the gut-liver axis theory, we believe that probiotics capable of regulating gut microbiota homeostasis represent a promising therapeutic strategy for effectively ameliorating NAFLD (16, 17). *Pediococcus acidilactici* (PA) has demonstrated beneficial effects in treating diabetes by modulating the gut microbiota and improving insulin resistance (18). Evidence from *Caenorhabditis elegans*, mouse, and rat models indicates that PA can regulate lipid metabolism and inflammatory responses, improve liver function and lower cholesterol, triglyceride and transaminase levels (19–22). Furthermore, studies suggest that probiotics may improve NAFLD progression by modulating the gut microbiota and inflammatory pathways (23, 24). Additionally, PA has shown beneficial effects in treating other conditions, including anxiety, constipation and neurodegenerative disorders (25–27).

Curcumin is used as the positive control, supported by established evidence demonstrating its efficacy in mitigating various chronic diseases, including cancer, diabetes, and NAFLD (28–30). Our research group has also demonstrated in a rat model that curcumin can improve NAFLD by modulating the gut microbiota and regulating the body’s inflammatory response (31).

In this study, we employed an animal model that closely mimics clinical realities where patients often struggle to maintain long-term dietary control by inducing NAFLD with a HFD followed by a recovery period and subsequent HFD re-challenge. This design provides a more accurate representation of PA53’s clinical application potential in real-world scenarios. By comparing PA53’s effects at different time points and against a curcumin control group, we demonstrate that early intervention (prevention) with PA53 is significantly superior to both therapeutic intervention and curcumin treatment, offering new insights for future NAFLD therapeutic strategies. Furthermore, this study provides novel perspectives on individualized NAFLD treatment, particularly regarding dietary interventions that leverage PA53’s natural presence in fermented foods (e.g., certain yogurts and pickled products), which is easily accessible for the public and provides a scientific basis for informed dietary choices, thereby reinforcing the translational potential of our findings.

## Materials and methods

### Strain

The *Pediococcus acidilactici* PA53 strain, a wild-type isolate derived from soybean tofu, is currently deposited in the China General Microbiological Culture Collection Center (CGMCC) under the conservation number CGMCC No. 18798. Colonies of PA53 are white, round and opaque, with a moist surface and smooth edges. It is Gram-positive, and microscopic examination revealed cells arranged in pairs. The probiotic strain PA53 was obtained from Wecare Probiotics Co., Ltd. (Suzhou, China) in the form of a lyophilized powder.

### Study design and animal experiments

All experimental procedures were conducted in accordance with the international guidelines for the care and use of laboratory animals and were approved by the Institutional Animal Care and Use Committee (IACUC) of the Shanghai Public Health Clinical Center (protocol number 2024-A007-01). Sixty-three male *C57BL/6J* mice aged 6 to 8 weeks (Hangzhou Ziyuan Experimental Animal Technology Co., Ltd.) were housed in the Specific Pathogen Free (SPF) area of the Animal Experimental Center of Shanghai Public Health Clinical Center. A 12-hour light-dark cycle was introduced to mimic natural conditions and support the circadian rhythm of the animals. The mice were fed a standard SPF diet with *ad libitum* access to sterile water.

**Figure 1:**
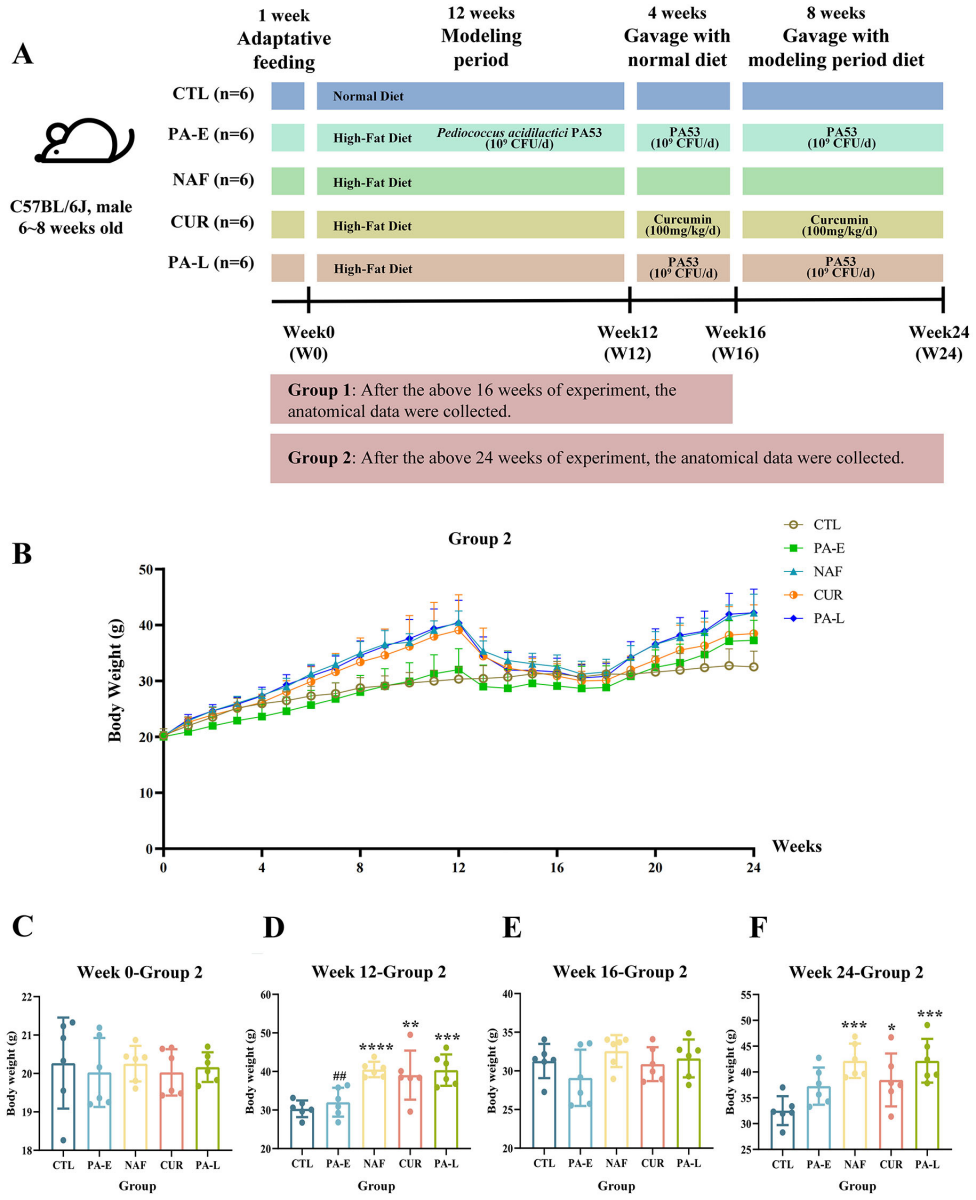
Experimental timeline and the effect of PA53 on body weight in a diet-cycling NAFLD model. **(A)** Schematic of the experimental design. **(B)** Body weight growth through the whole period. **(C)** Body weight of group 2 at week 0. **(D)** Body weight of group 2 at week 12. **(E)** Body weight of group 2 at week 16. **(F)** Body weight of group 2 at week 24. CTL, control group; PA-E, *Pediococcus acidilactici* PA53 early prevention group; NAF, NAFLD group; CUR, curcumin group; PA-L, *Pediococcus acidilactici* PA53 late treatment group. Values were expressed as mean ± SD (n = 6). *p < 0.05, **p < 0.01, ***p < 0.001, ****p < 0.0001 compared to the CTL group. ^##^p < 0.01 compared to the NAF group.

The experimental design, including mouse grouping, dietary interventions, treatment timelines, and sample collection procedures, is illustrated in **Figure 1A**. After a one-week acclimatization period, the mice were divided into five groups: CTL group (control group), PA-E group (PA53 early prevention group), NAF group (NAFLD group), CUR group (curcumin group) and PA-L group (PA53 late treatment group). For the first 12 weeks, all groups except CTL received a high-fat diet (HFD, 60% kcal fat, Research Diets D12492) (32). The PA-E group also received oral PA53 (1×10^9^ CFU/d) for early intervention. At week 12, one mouse from CTL, PA-E, and NAF groups was randomly selected for liver hematoxylin and eosin (H&E) staining to assess model efficacy alongside body weight. From week 12 to week16, all mice were switched to a normal diet. Pharmacological treatments were administered daily via oral gavage: mice in the PA-E and PA-L groups received PA53 at a dose of 1×10^9^ CFU suspended in 0.2 mL of 0.5% sodium carboxymethylcellulose (NaCMC, C8620, Solarbio) per mouse; mice in the CUR group received curcumin (Jiaherb, Xi’an) at 100 mg/kg body weight in the same vehicle. To control for the gavage procedure, mice in the CTL and NAF groups received an equal volume (0.2 mL) of the 0.5% NaCMC vehicle. Each group consisted of two subgroups (group 1 and group 2, n=6), and anatomical samples were collected from 30 mice in group 1 at week 16 and group 2 at week24. The sample size was determined based on common practice in murine NAFLD models with relevant interventions (33–35), and is consistent with previous studies reporting robust statistical power for similar outcome measures.

The animal experiment lasted 24 weeks, with mice’s body weights recorded weekly. At the end of the study (week 16 for group 1, week 24 for group 2), mice fasted for 12 hours were anesthetized with 2-5% isoflurane prior to tissue collection and euthanized via cervical dislocation, ensuring unconsciousness before death. And animal welfare was monitored rigorously throughout. Blood was collected via the orbital sinus, and serum was collected by centrifugation at 1000 × g for 15 minutes at 4°C and then stored at -80°C for subsequent analysis. Liver and ileum tissues from the designated areas of the mice were fixed in 4% paraformaldehyde for subsequent histopathologic dissection and staining. Fecal samples were collected at weeks 0, 12, 16 and 24, rapidly frozen with liquid nitrogen and stored at -80°C.

### Hematology detection

Serum samples were collected from mice at two time points, W16 and W24. Serum concentrations of alanine aminotransferase (ALT), glucose (GLU), triglyceride (TG), cholesterol (CHO) and low-density lipoprotein cholesterol (LDL-C) were measured using an automatic biochemical analyzer (Chemray 800, China). Serum concentrations of inflammatory cytokines, including tumor necrosis factor-α (TNF-α), interleukin-1β (IL-1β), interleukin-6 (IL-6), and interferon-inducible protein-10 (IP-10), were determined using ELISA kits (Jingmei Biotechnology, Jiangsu, China) according to the manufacturer’s instructions.

### Histological staining of liver and intestinal tissues

Liver and ileum samples from the same anatomical location in each mice were fixed in 4% paraformaldehyde. Following fixation, the samples were dehydrated (Shandon Excelsior AS, Thermo), embedded (Shandon Histostar, Thermo) and cut into 3 µm thick kerosene sections (Leica RM2245). Sections were then deparaffinized, stained with H&E, dehydrated and mounted before microscopic examination. Liver H&E staining results were subjected to semiquantitative analysis using the NAFLD activity score (NAS), which assesses steatosis, hepatocellular ballooning, and inflammation. To further investigate lipid deposition in liver tissue, liver samples from the same anatomical site were embedded in OCT and stained with Oil Red O. Quantitative analysis of the staining results was performed using ImageJ (version 1.54g, National Institutes of Health, USA) (36, 37).

### Utilizing 16S rRNA sequencing for microbial profiling

16S rRNA was conducted on fecal samples collected from 30 mice of group 2 at three time points: weeks 0, 12, and 24. DNA extraction from the fecal samples was carried out using the E.Z.N.A. Stool DNA Kit (Omega Bio-tek, Inc., GA). The V3-V4 region of the 16S rRNA gene was amplified by PCR using the following primers: Forward primer 5’-CCTACGGGNGGCWGCAG-3’ and Reverse primer 5’-GACTACHVGGGTATCTAATCC-3’. PCR amplification was performed on an EasyCycler 96 PCR system (Analytik Jena Corp., AG) under the following conditions: an initial denaturation at 95°C for 3 minutes, followed by 21 cycles of denaturation at 94°C for 30 seconds, annealing at 58°C for 30 seconds and extension at 72°C for 30 seconds, with a final extension at 72°C for 5 minutes. The amplified DNA fragments were then subsequently using the MiSeq platform (Illumina Inc., USA).

### In silico analysis​​

Following sequencing, raw paired-end reads were processed to remove primer sequences and low-quality bases using cutadapt (v2.6). Standard quality filtering was applied, retaining reads with a length between 400 and 500 bp. The high-quality sequences were then dereplicated and clustered into Operational Taxonomic Units (OTUs) at a 97% similarity threshold using the UPARSE algorithm within USEARCH (v11.0), which simultaneously identifies and removes chimeric sequences. For taxonomic annotation, a representative sequence from each OTU was classified using the Ribosomal Database Project (RDP) Classifier (v2.13) against the SILVA database (Release 138). All downstream analyses were performed using QIIME (v1.9.1). α-diversity, encompassing richness (Chao1, ACE) and diversity (Shannon, Simpson) indices, was calculated. β-diversity was assessed using Bray-Curtis dissimilarity and visualized via principal coordinate analysis (PCoA). Statistical differences in overall microbial community structure between groups were tested using permutational multivariate analysis of variance (PERMANOVA).

### Statistical analysis

Statistical analysis was performed using SPSS Statistics 20.0 (IBM Corp., Armonk, NY, USA). The Kruskal-Wallis test was used to assess differences in the overall distribution of samples among groups. Pairwise comparisons between groups were also performed using the Wilcoxon rank sum test (Mann-Whitney U test). P-values from these comparisons were corrected using the Bonferroni method. A p-value less than 0.05 was considered statistically significant. The datasets generated during the current study are available in the NCBI genome database repository (PRJNA1219805).

## Results

### Changes in body weight parameters of mice

Body weight was recorded weekly throughout the study. The data were plotted as a line graph (**Figure 1B**) and differences in body weight at key time points (W0, W12, W16, W24) were analyzed for each group. At W0, no significant differences in body weight were found between the groups (p > 0.05, **Figure 1C**). After a 12-week high-fat diet, during which probiotics were administered to the PA-E group for prevention, the significant differences in body weight were evident in all groups compared to the CTL group (p < 0.01 for all), except for the PA-E group (p > 0.05). Furthermore, the body weight of the PA-E group showed a significant difference compared to the NAF group (p = 0.0045, **Figure 1D**). Following a four-week period on a normal diet, no significant differences in body weight were observed between the groups (p > 0.05, **Figure 1E**). The weight changes in group 1 showed a similar trend to group 2 in the first 16 weeks mentioned above (**Supplementary Figure S1A**). During the final eight weeks in group 2, all mice, except the CTL group, were switched back to a high-fat diet. Body weight analysis revealed no significant differences between the PA-E groups compared to the CTL group (p = 0.1384), while the NAF, CUR and PA-L groups showed significant differences compared to the control group (p=0.0009, p = 0.0464, and p=0.0009, respectively; **Figure 1F**).

### Pathological staining results of the liver and ileum

**Figure 2:**
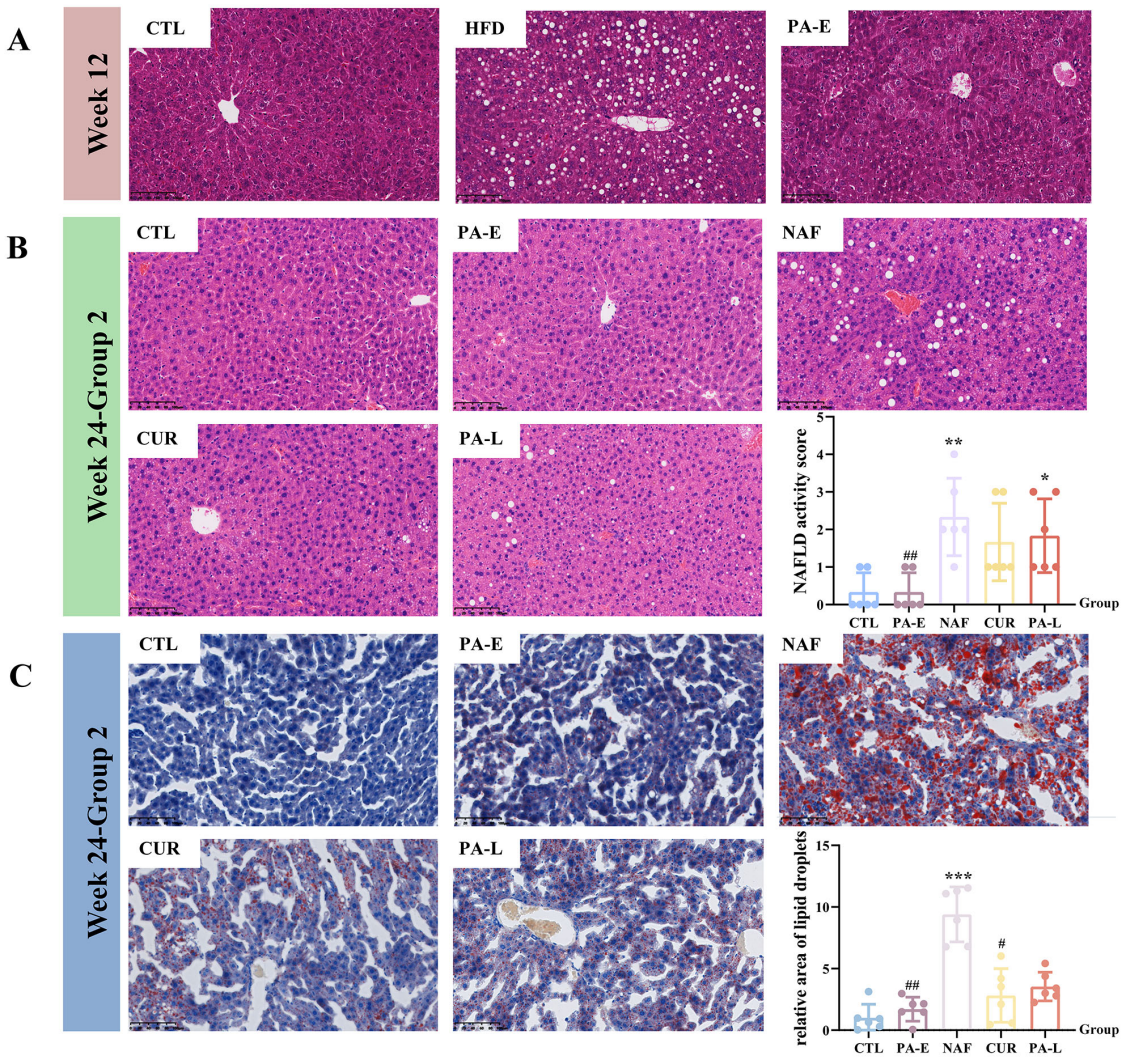
H&E staining and Oil red O staining of mouse livers (magnification × 200). **(A)** H&E staining images of liver sections from mice at Week 12. **(B)** H&E staining images of liver sections from mice at Week 24. **(C)** Oil red O staining images of liver sections from mice at Week 24. CTL, Control group; HFD, High-Fat Diet group; PA-E, *Pediococcus acidilactici* PA53-Early prevention group; NAF, NAFLD group; CUR, Curcumin group; PA-L, *Pediococcus acidilactici* PA53-Late treatment group. Values were expressed as mean ± SD (n = 6). *p < 0.05, **p < 0.01, ***p < 0.001 compared to the CTL group. ^#^p < 0.05, ^##^p < 0.01 compared to the NAF group.

At W12, following HFD modeling, one mouse each from the CTL, PA-E and NAF groups was randomly selected for H&E staining of the liver. The results showed significant fat deposition in the HFD group, while no conspicuous fat vacuoles were detected in the CTL and PA-E groups (**Figure 2A**). Consistent with the body weight data at W12, this indicated successful establishment of the fatty liver model. Four weeks after drug administration (at W16), mice in group 1 were euthanized for tissue collection, and liver tissue from all groups was stained with H&E. H&E staining revealed no conspicuous fat vacuoles in any group, and the microscopic morphology resembled normal liver tissue(**Supplementary Figure S1B**). Subsequently, the mice in group 2 underwent an eight-week intervention that included a high-fat diet combined with drug treatment, and the tissues were harvested at W24. Liver tissues were subjected to H&E and Oil Red O staining, followed by corresponding semi-quantitative and quantitative analyzes. Compared to the CTL group, the NAF group exhibited significant fat deposition. The PA-E group displayed a pathologic staining pattern similar to the CTL group. Both the CUR and PA-L groups demonstrated some improvement in fat deposition, though less pronounced than in the PA-E group (**Figures 2B, C**).

**Figure 3:**
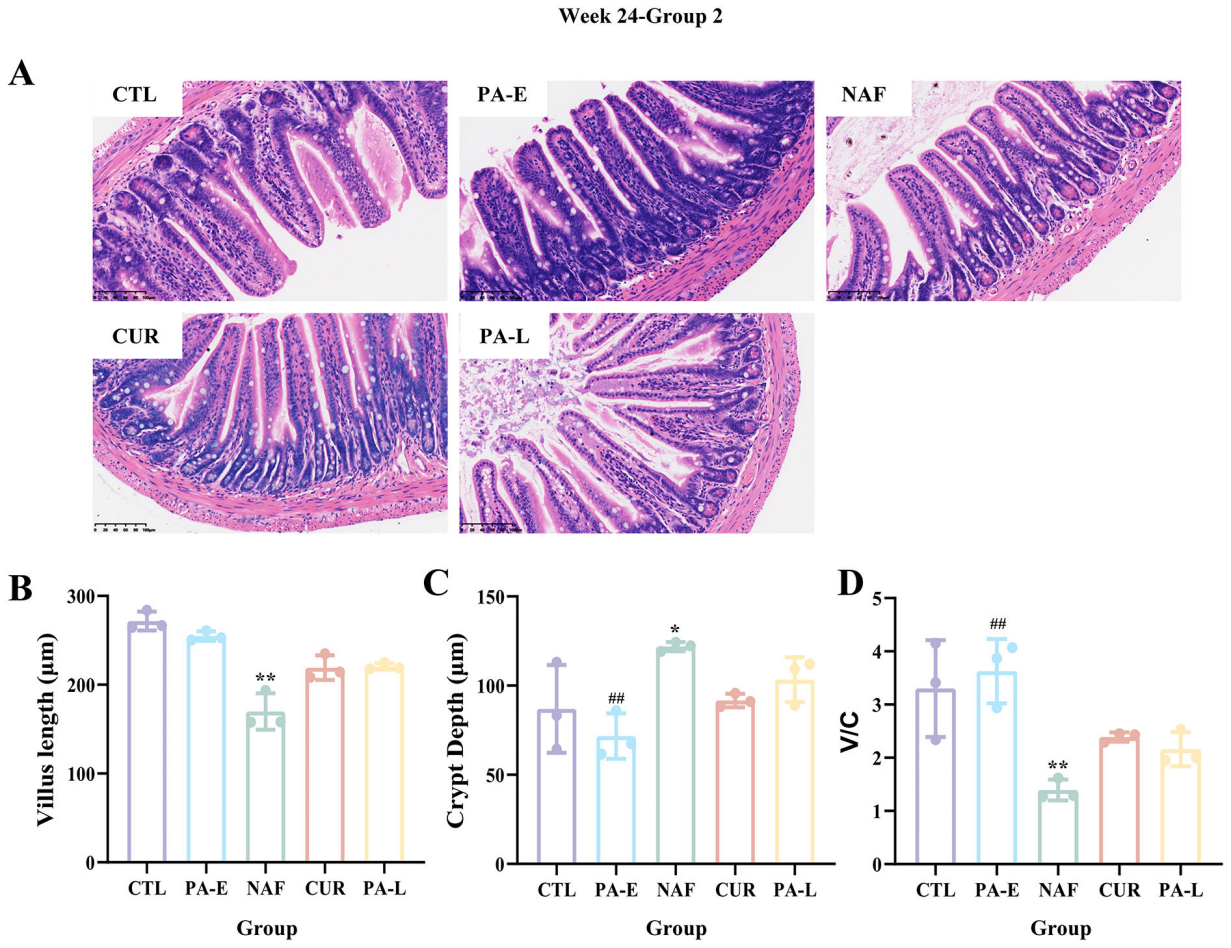
H&E staining of mouse ileum and analysis of intestinal morphology (magnification × 200). **(A)** Representative H&E staining images of ileum sections from different groups at Week 24, showing villus structure. **(B)** Bar graph showing the villus length (μm) of each group. **(C)** Bar graph showing the crypt depth (μm) of each group. **(D)** Bar graph showing the villus/crypt ratio (V/C) of each group. CTL, Control group; HFD, High-Fat Diet group; PA-E, *Pediococcus acidilactici* PA53-Early prevention group; NAF, NAFLD group; CUR, Curcumin group; PA-L, *Pediococcus acidilactici* PA53-Late treatment group. Values were expressed as mean ± SD (n = 3). *p < 0.05, **p < 0.01. ^##^p < 0.01 compared to the NAF group.

The ileal tissue of the mice in group 1 and group 2 was subjected to H&E staining. Microscopic examination revealed that the tissue maintained its structural integrity and the cells showed a regular and healthy morphology. At time points W16, there were no significant differences in the length of small intestinal villi between all groups (**Supplementary Figure S1C**). At time points W24, compared with the CTL group, the NAF group showed a significant decrease in villus length (p = 0.0041) and a significant increase in crypt depth (p = 0.0346), accompanied by a significant decrease in the villus to crypt ratio (V/C, p = 0.0038), while the PA-E group could effectively reverse this trend (**Figure 3**).

### Modulation of serum inflammatory markers and biochemical parameters

**Figure 4:**
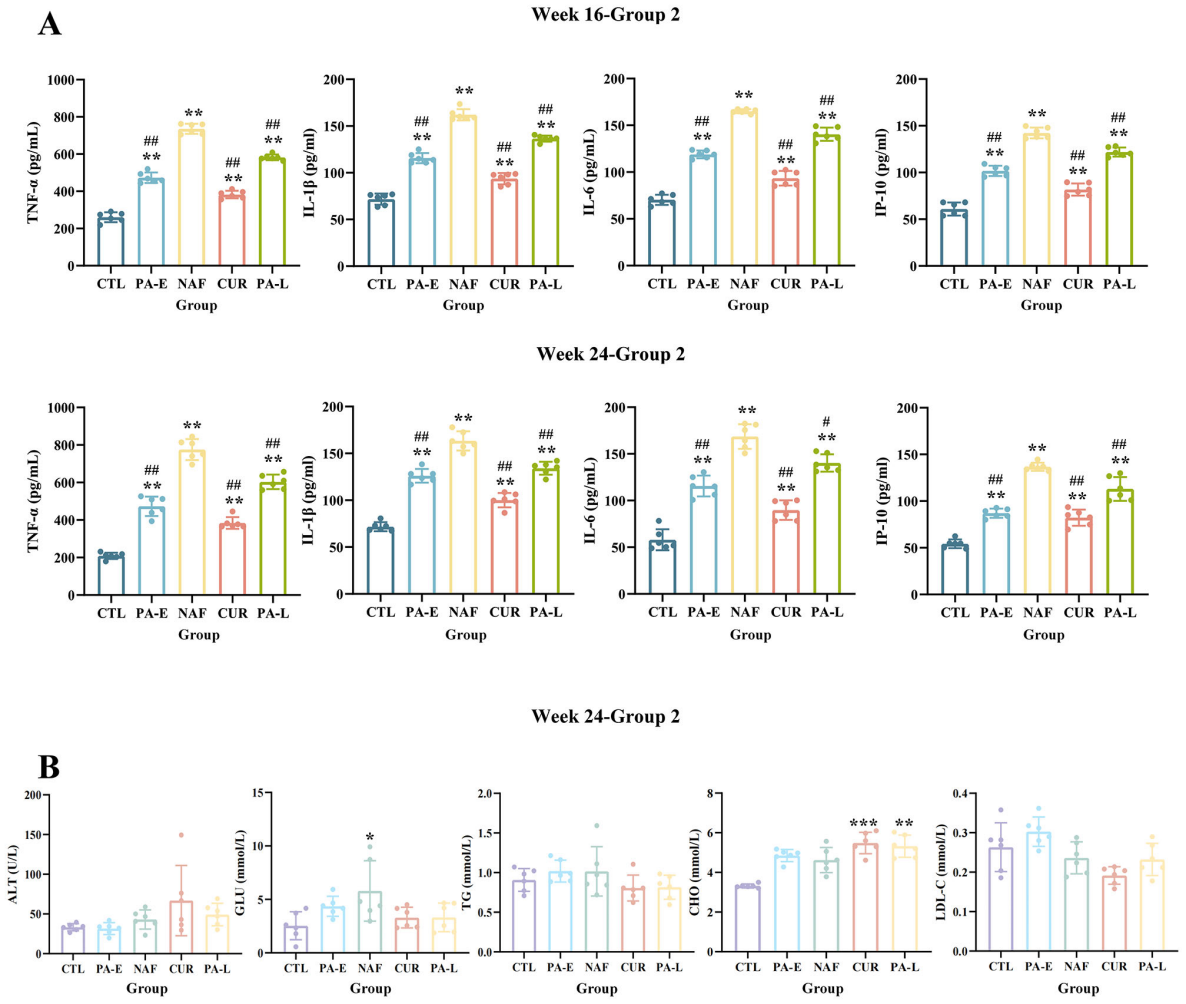
Serum inflammatory factor levels and biochemical parameters in mice after different treatments. **(A)** Serum levels of inflammatory factors (TNF-α, IL-1β, IL-6, and IP-10) measured by ELISA at Week 16 and Week 24. **(B)** Biochemical blood test results (ALT, GLU, TG, CHO, and LDL-C) at Week 24. CTL: control group, PA-E: *Pediococcus acidilactici* PA53 early prevention group, NAF: NAFLD group, CUR, curcumin group; PA-L, *Pediococcus acidilactici* PA53 late treatment group. TNF-α, tumor necrosis factor-α; IL-1β, interleukin-1β; IL-6, interleukin-6; IP-10, interferon-inducible protein-10; ALT, alanine aminotransferase; GLU, glucose; TG, triglycerides; CHO, cholesterol; LDL-C, low-density lipoprotein cholesterol. Values were expressed as mean ± SD (n = 6). *p < 0.05, **p < 0.01, ***p < 0.001 compared to the CTL group. ^#^p < 0.05, ^##^p < 0.01 compared to the NAF group.

Levels of inflammatory factors, including TNF-α, IL-1β, IL-6 and IP-10, were measured in group 1 mice at week 16 (W16) (**Supplementary Figure S1D**) and in group 2 mice at both W16 and week 24 (W24) (**Figure 4A**). Compared to the CTL group, the NAF group exhibited significantly elevated levels of all measured inflammatory factors. In contrast, both PA53 and CUR treatments effectively attenuated the HFD-induced increase in these inflammatory factors (p < 0.01). Serum biochemical parameters, namely ALT, TG, CHO and LDL-C, were also assessed for both group 1 and group 2. While some variation in these parameters were observed across the groups, no statistically significant differences were detected between any of the groups in group 1 (**Supplementary Figure S1E**). Group 2 shows a similar trend, but the intervention group has a certain protective effect on GLU (**Figure 4B**).

### Effects of the PA53 intervention on gut microbiota diversity

**Figure 5:**
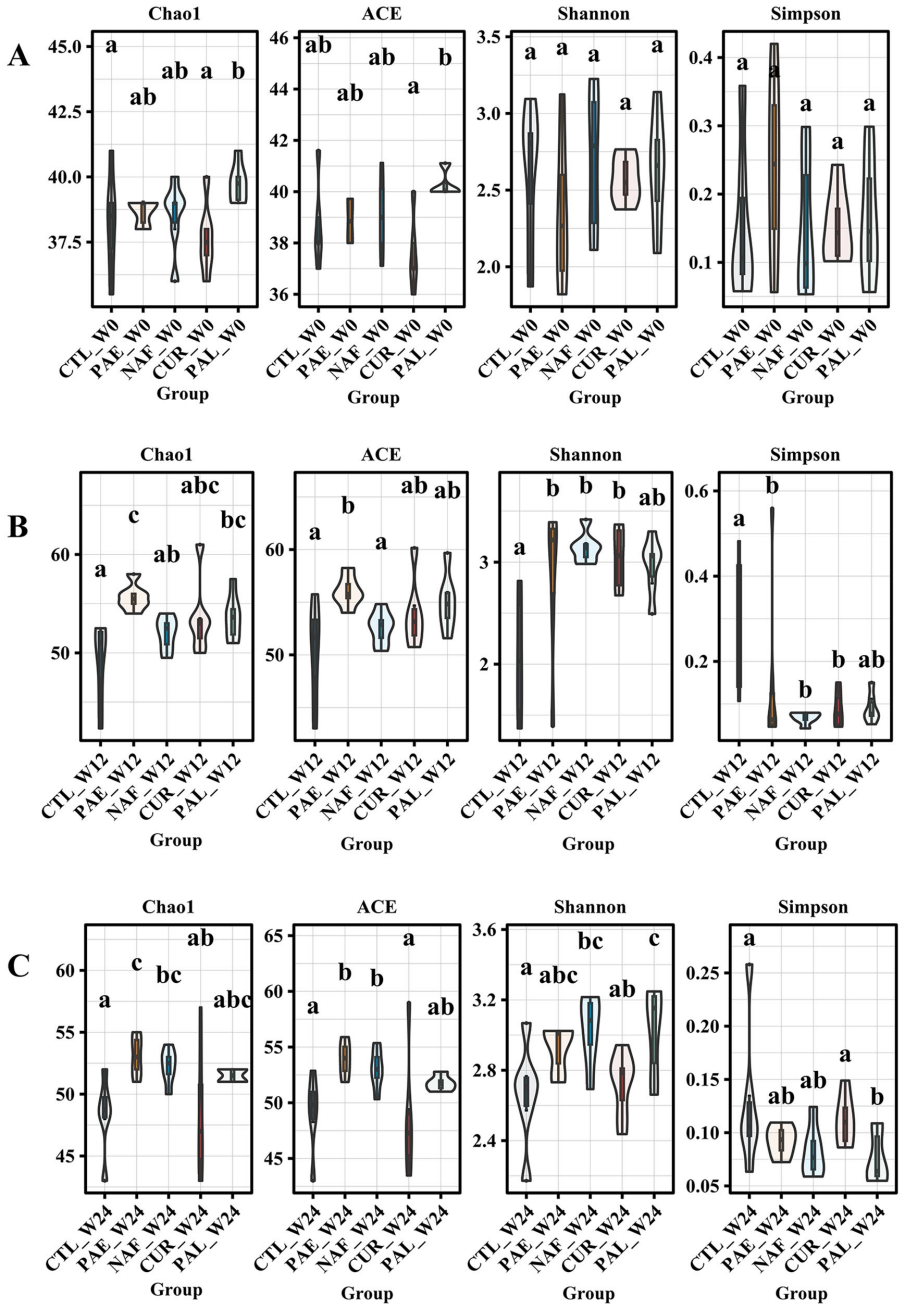
Alpha-diversity indices of gut microbiota at different time points. **(A)** Alpha-diversity metrics (Chao1, ACE, Shannon, and Simpson indices) of fecal samples at week 0 (W0). **(B)** Alpha-diversity metrics at week 12 (W12). **(C)** Alpha-diversity metrics at week 24 (W24). CTL, control group; PA-E, *Pediococcus acidilactici* PA53 early prevention group; NAF, NAFLD group; CUR, curcumin group; PA-L, *Pediococcus acidilactici* PA53 late treatment group. All the averages are listed in order from smallest to largest, and then the smallest average is marked with the letter a; This average is compared with the following averages, where the difference is not significant, marked with the letter a, until a certain average with a significant difference, marked with the letter b Where there is an identical marking letter, the difference is not significant, and where there is a different marking letter, the difference is significant (p<0.05).

At W0, significant differences in Chao1 and ACE indices were found between certain groups, which might be attributed to the increase of in certain low-abundance taxa potentially leading to higher estimated species richness. In contrast, no significant differences were found in the Shannon and Simpson indices, indicating comparable α-diversity across the groups. Following 12 weeks of high-fat diet, α-diversity indices exhibited varying degrees of change in all groups relative to the CTL group. By W24, post-drug intervention, the α-diversity indices had partially recovered in all groups, with their values approaching those of the CTL group (**Figure 5**).

**Figure 6:**
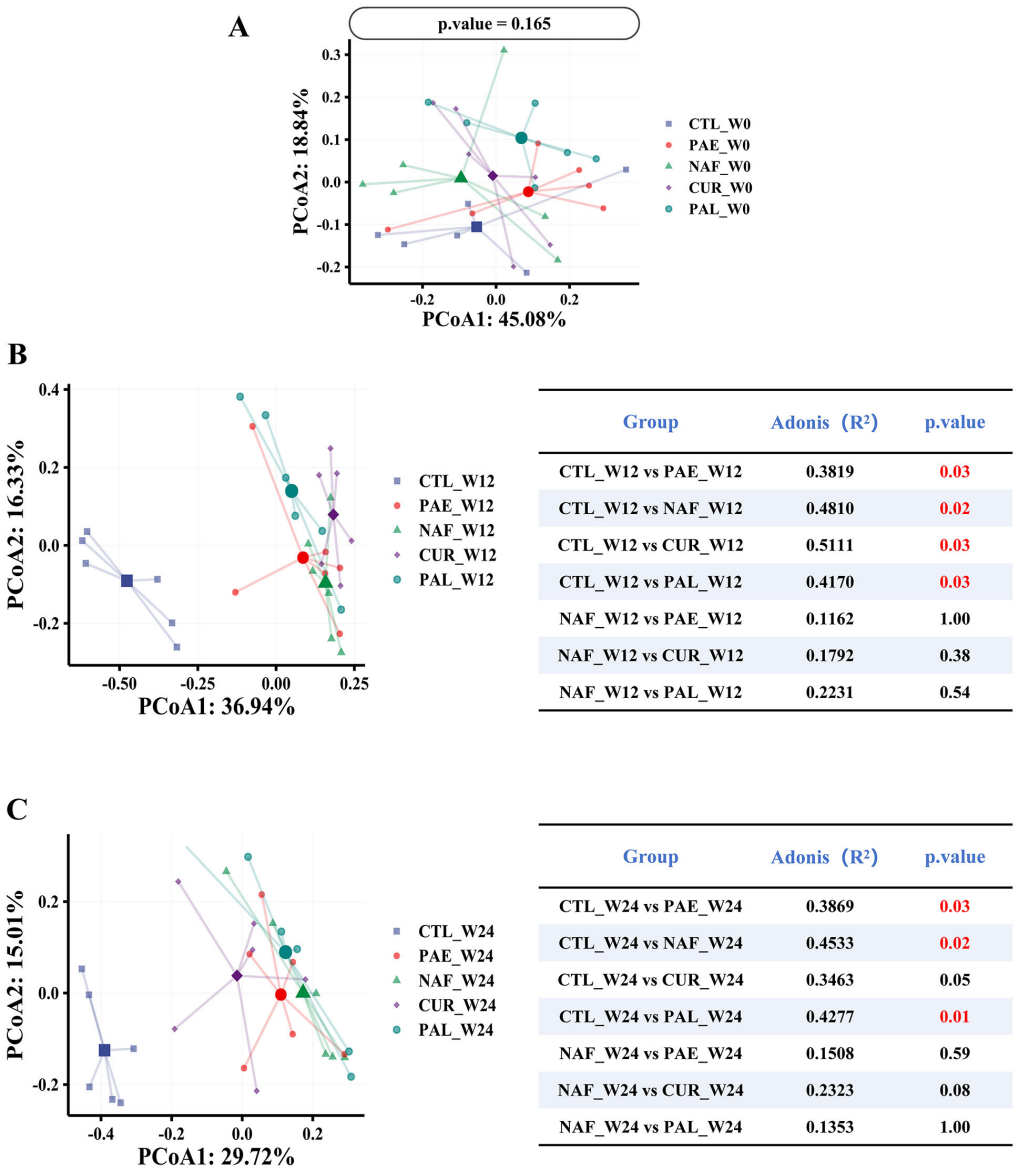
β-diversity analysis of gut microbiota at different time points. **(A)** Principal coordinate analysis (PCoA) of gut microbiota at week 0 (W0). **(B)** PCoA plot at week 12 (W12). **(C)** PCoA plot at week 24 (W24). CTL, control group; PA-E, *Pediococcus acidilactici* PA53 early prevention group; NAF, NAFLD group; CUR, curcumin group; PA-L, *Pediococcus acidilactici* PA53 late treatment group.

PCoA based on Bray-Curtis distance was employed to evaluate the changes in β-diversity in the gut microbiota, with each point representing an individual mouse’s microbiota composition. The Adonis method was used to determine the statistical significance of communities differences between groups. At W0, no significant overall group differences were observed (p = 0.165). After a 12-week high-fat diet, significant differences were found between all groups and the CTL group, whereas no significant differences were found between the groups and the NAF group. Following drug intervention, no significant difference was observed between the CUR group and the CTL group at W24, while significant differences (p < 0.05) persisted between the other groups and the CTL group (**Figure 6**).

### Analysis of the gut microbiota at phylum and genus levels

**Figure 7:**
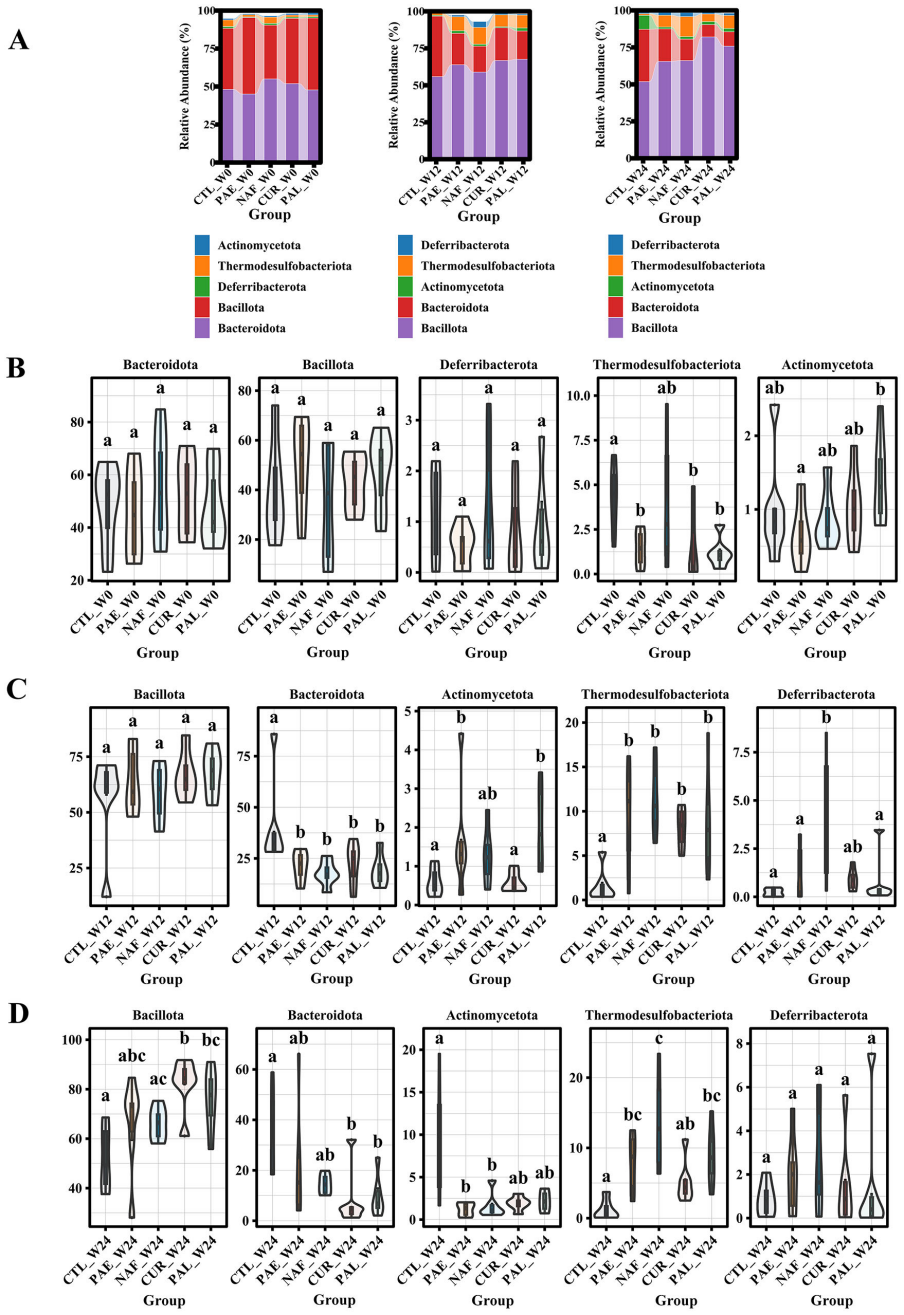
Analysis of gut microbiota composition at different time points. **(A)** Stacked bar charts showing the relative abundance of bacterial phyla at week 0 (W0), week 12 (W12), and week 24 (W24). **(B-D)** Box plots depicting the relative abundance of specific phyla (*Bacteroidota, Bacillota, Deferribacterota, Thermodesulfobacteriota, Actinomycetota*) at W0 **(B)**, W12 **(C)**, and W24 **(D)** CTL, control group; PA-E, *Pediococcus acidilactici* PA53 early prevention group; NAF, NAFLD group; CUR, curcumin group; PA-L, *Pediococcus acidilactici* PA53 late treatment group. All the averages are listed in order from smallest to largest, and then the smallest average is marked with the letter a; This average is compared with the following averages, where the difference is not significant, marked with the letter a, until a certain average with a significant difference, marked with the letter b Where there is an identical marking letter, the difference is not significant, and where there is a different marking letter, the difference is significant (p<0.05).

Across all time points (W0, W16, and W24), the top five phyla in the mouse gut microbiota consistently comprised *Bacteroidota*, *Bacillota*, *Deferribacterota*, *Thermodesulfobacteriota*, and *Actinomycetota*. Specifically, *Bacteroidota* was the most abundant phylum at W0, while *Bacillota* was the most abundant at W16 and W24, as visualized in the relative abundance plots (**Figure 7A**). Compared to W0, the high-fat diet (W12) resulted in a significant decrease in *Bacteroidota* and a significant increase in *Thermodesulfobacteriota* in all groups. At W24, the relative abundance of *Bacteroidota* was increased in the PA-E group, with no significant difference compared to the CTL group. The CUR group showed a decrease in *Thermodesulfobacteriota*, with a significant difference compared to the NAF group (**Figures 7B–D**).

At the genus level, microbiota analysis revealed that both curcumin and PA53 contributed to maintaining gut microbiota stability and promoting the abundance of certain beneficial bacteria. At W0, overall gut microbiota profiles showed no significant differences among the groups. At week 12, the CUR, NAF and PA-L groups were all HFD-induced NAFLD mice, while the PA-E group received early intervention with PA53. Early PA53 intervention effectively mitigated HFD-induced alterations in the abundance of *Romboutsia* and *Lactococcus*, bringing their levels in the PA-E group closer to those of the CTL group.

Genus-level microbiota analysis at W24 also revealed similar trends. Gut microbiota analysis demonstrated a significant increase in *Pediococcus* in the PA-E group compared to the other groups, suggesting that the oral PA53 intervention is reliable and has the potential to colonize the mouse gut. Probiotics and curcumin supplementation improved the abundance of *Rikenella*, *Blautia*, *Roseburia* and *Colidextribacter*, which had been altered by the HFD. Notably, curcumin exerted a significant regulatory effect on *Rikenella* and *Roseburia*, PA-L significantly affected *Rikenella* and PA-E prominently influenced *Roseburia*. Although the PA-E group did not show a significant difference in *Adlercreutzia* regulation, compared to CUR and PA-L groups, its levels were numerically lower. Compared to the other groups, the PA53 intervention groups (PA-E and PA-L) showed increased levels of *Lachnospiraceae* NK4A136 and *Pediococcus* (**Supplementary Table 1**).

## Discussion

Our study demonstrates that PA53 exerts beneficial effects on controlling body weight, improving hepatic lipid deposition, regulating the body’s inflammatory response and maintaining the stability of the gut microbiota. PA53 can effectively prevent and delay the onset and progression of NAFLD in mice. Additionally, preventive treatment administered early before disease onset proved more effective than treatment initiated at later stages.

Notably, in a separate experiment (group 1), we confirmed that the pathological features of NAFLD were rapidly reversed upon switching to a normal diet, underscoring the fundamental importance of dietary intervention (7, 38–41). Nevertheless, in clinical practice, numerous patients are unable to strictly adhere to a controlled diet. Therefore, to mimic the real-life situation in which patients are unable to fully control their diet, we formed another experimental group. In group 2, PA-E and CUR effectively reduced weight gain from the high-fat diet. Liver pathology showed the PA-E group’s histology most closely resembled the CTL group, significantly differing from the NAF group at week 24. Both CUR and PA-L also reduced lipid deposition compared to NAF, though less effectively than PA-E.

Our findings on the efficacy of PA53 in ameliorating HFD-induced NAFLD align with and substantially extend the growing body of evidence supporting the metabolic benefits of the *Pediococcus acidilactici* species. Previous studies have established that *P. acidilactici* strains can improve glucose homeostasis, dyslipidemia, and systemic inflammation in rodent and *C. elegans* models of obesity and metabolic disorder (18–22, 42–44). However, our study offers key conceptual and design advances. First, unlike previous studies using continuous HFD-feeding models (21, 22, 42, 44), we employed a diet-cycling model with HFD re-challenge. PA53 sustained protection against hepatic steatosis during this second HFD phase, demonstrating its resilience in a clinically relevant context of dietary fluctuation. Second, while prior probiotic administration coincided with HFD onset, we systematically compared preventive (PA-E) versus therapeutic (PA-L) regimens. The clear superiority of early administration establishes that PA53’s efficacy critically depends on intervention prior to significant dysfunction, positioning it as a viable preventive agent and advancing the understanding of the probiotic therapeutic window. Finally, as *P. acidilactici* is native to fermented foods (20, 45), our work provides a rationale for leveraging dietary probiotics as a feasible, population-level strategy for NAFLD risk reduction, bridging experimental findings with public health translation.

Previous studies have shown that supplementation with probiotics can effectively improve levels of inflammatory cytokines such as TNF-α, IL-6 and IL-1β in NAFLD mouse or rat models (23, 46–51). Our results align with prior studies. Serum concentrations of TNF-α, IL-1β, IL-6, and IP-10 were assessed at weeks 16 and 24, with significant intergroup variations observed at both timepoints. Among the different treatments, curcumin yielded the strongest anti-inflammatory effect. PA53 likewise showed anti-inflammatory potential, and notably, early probiotic prevention resulted in a more significant reduction in inflammation compared to late treatment. Probiotics are reported to positively influence liver function and reduce triglyceride and cholesterol levels (23, 47, 48), a finding supported by clinical studies. For instance, supplementation with *Bifidobacterium longum* or mixed probiotic formulations can significantly reduce body mass index (BMI) and serum concentrations of inflammatory cytokines in NAFLD patients (52, 53). However, conflicting results exist: a six-month RCT in 48 NASH patients found that probiotics alone did not significantly improve the elevated levels of inflammatory cytokines or transaminases (54). Such discrepancies may stem from variations in disease severity, interspecies differences, or the specific probiotic strains and combinations used. Further animal and clinical research is needed to fully validate probiotics’ efficacy and elucidate their mechanisms.

In this study, we observed that the hepatocellular injury marker ALT did not exhibit a significant increase in the high-fat diet-induced model group (NAF), a finding consistent with previous reports (55). ALT, an enzyme primarily located within hepatocytes, is released into the bloodstream upon cellular damage, leading to elevated serum concentrations (56). The phenotype of elevated serum inflammatory cytokines without a concurrent rise in ALT aligns with the “multiple hits hypothesis” of MAFLD progression, which posits that metabolic inflammation is a primary early event that can precede overt hepatocellular necrosis (57, 58). Furthermore, our study revealed concomitant intestinal barrier damage and alterations in gut microbiota composition in the NAF group, suggesting that the observed systemic inflammation likely originates from the translocation of gut-derived bacterial products, such as lipopolysaccharide (LPS), into the liver, leading to sustained immune cell activation (16). This observation mirrors the clinical reality where a substantial proportion of MAFLD patients present with normal serum ALT levels despite having confirmed hepatic inflammation and fibrosis (59–61). Therefore, rather than being a contradiction, the dissociation between elevated inflammatory markers and stable ALT in our model is a critical finding. It highlights the potential mechanism by which the probiotic PA53 may prevent liver injury by modulating the gut-liver axis and intervening in metabolic inflammation during the crucial early stages of the disease, thereby holding significant clinical implications.

The analysis of α-diversity provides insights into the within-sample complexity of the gut microbiota. At baseline (W0), comparable Shannon and Simpson indices across all groups confirmed a similar initial community structure, validating our experimental design, despite some variations in Chao1/ACE indices likely due to low-abundance taxa. As expected, the HFD altered α-diversity by W12. Critically, by W24, interventions with PA53 and curcumin prompted a partial recovery of these indices, mitigating the HFD-induced loss of microbial diversity. However, this recovery was incomplete, and β-diversity remained distinct. This partial restoration of α-diversity likely contributed to the observed metabolic improvements, while the limited impact on β-diversity may be attributed to the intervention duration.

PA53 demonstrated the potential to colonize the gut and modulate the gut microbiota composition. At both W12 and W24, genus-level analysis revealed a significant increase in PA53 abundance within the intervention group compared to controls, confirming the efficacy of gavage administration. Analysis at both phylum and genus level further indicate that early PA53 intervention effectively maintains gut microbiota homeostasis and enhances the abundance of specific beneficial bacteria, such as *Lachnospiraceae* NK4A136. Given that *Lachnospiraceae* NK4A136 influences liver metabolism, inflammation and lipid accumulation via short-chain fatty acids production (62), this bacterium may represent a key mediator through which PA53 regulates NAFLD via the gut-liver axis.

**Figure 8:**
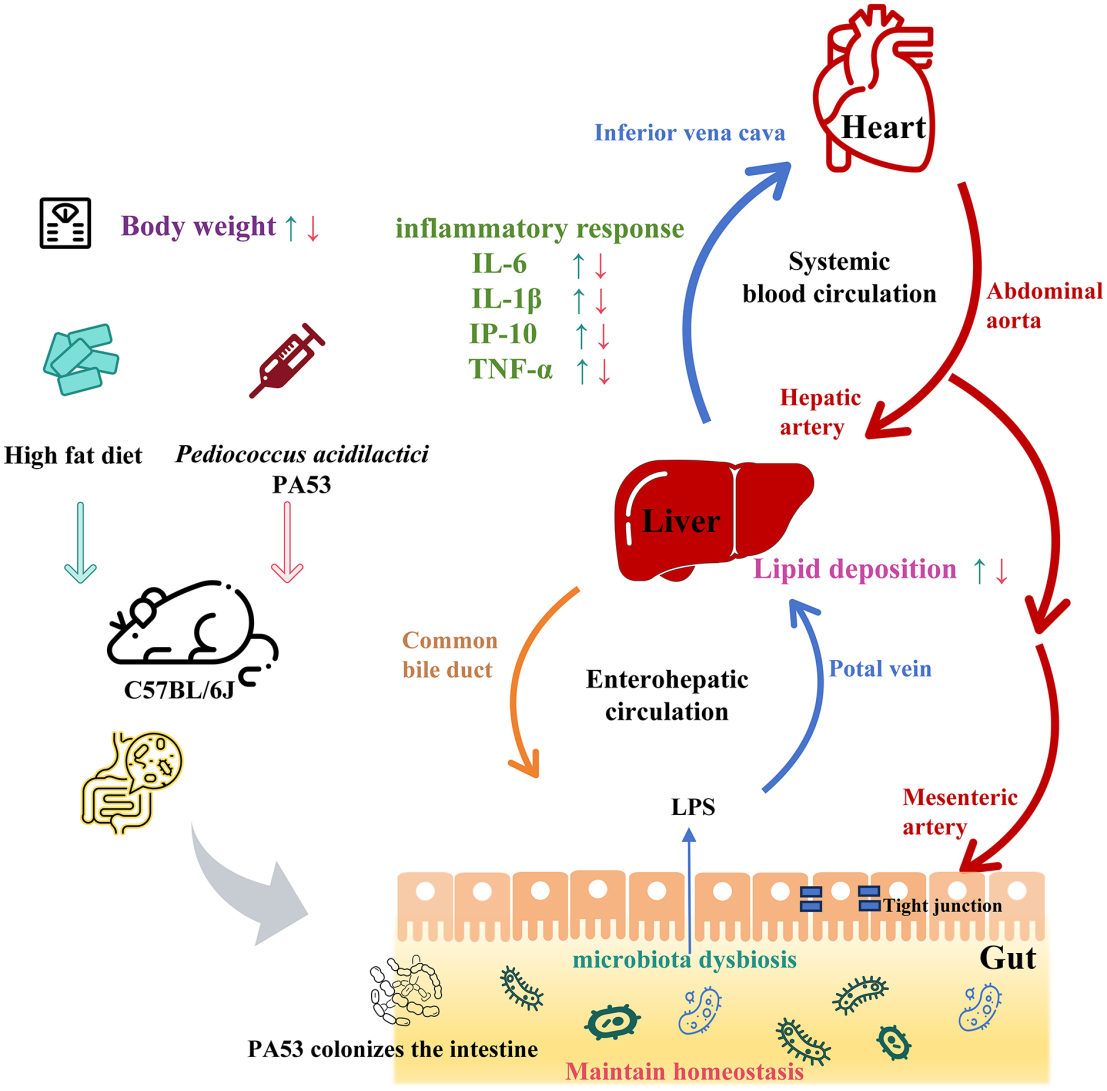
Possible mechanism of action of *Pediococcus acidilactici* PA53. TNF-α, tumor necrosis factor-α; IL-1β, interleukin-1β; IL-6, interleukin-6; IP-10, interferon-inducible protein-10.

A high-fat diet disrupts the homeostasis of gut microbiota and impairs intestinal barrier integrity, thereby facilitating the translocation of gut-derived endotoxins—such as lipopolysaccharides (LPS)—into the systemic circulation (63, 64). Circulating LPS subsequently activates systemic immune responses, triggering the release of pro-inflammatory cytokines including IL-6, IL-1β, TNF-α, and IP-10 in the serum. These pro-inflammatory mediators further promote hepatic lipid accumulation and insulin resistance, directly driving the initiation and progression of NAFLD (65, 66). This well-characterized endotoxin-mediated signaling pathway provides a compelling mechanistic framework to interpret our findings (**Figure 8**). Although we did not measure LPS levels directly, the marked reduction in hepatic inflammation observed following PA53 treatment is highly consistent with the restoration of intestinal barrier function and a subsequent decrease in endotoxin influx into the circulation. Collectively, our results establish a plausible mechanistic link, whereby PA53 exerts its NAFLD-ameliorating effects, at least in part, by interrupting this deleterious gut-liver axis communication. PA53 supplementation effectively normalized body weight, hepatic lipid accumulation, serum pro-inflammatory cytokine levels, and gut microbiota composition in our model. Notably, the preventive PA53 treatment group exhibited superior improvements in NAFLD-related phenotypes compared with the delayed treatment group, though the specific molecular mechanisms underlying this differential therapeutic efficacy warrant further investigation.

In summary, while our study demonstrates the beneficial effects of PA53 in a dietary reversal model of NAFLD, we acknowledge several important limitations that warrant consideration and point to future research directions. Primarily, a larger sample size is warranted for more robust and generalizable conclusions. Second, although our dietary model was designed to simulate clinical conditions, incorporating a continuous HFD group and an additional cohort receiving PA53 monotherapy under a normal diet would further validate the specific efficacy of PA53. Additionally, future mechanistic studies employing more diverse fatty liver models are needed to clarify PA53’s specific actions. Furthermore, future studies should employ functional tests such as pyruvate Tolerance Test (PTT) and insulin tolerance tests to precisely delineate the effects of PA53 on hepatic gluconeogenesis and systemic insulin resistance, thereby providing a more comprehensive understanding of its impact on glucose metabolism. Addressing these points in future studies will be crucial to fully elucidate the therapeutic potential and mechanism of action of PA53.

## Conclusion

Our study demonstrates that early intervention with the probiotic strain *Pediococcus acidilactici* PA53 is superior to therapeutic treatment in preventing the onset and progression of NAFLD. Utilizing a clinically relevant diet-cycling model, we found that preventive administration of PA53 effectively averted hepatic steatosis and weight gain. These protective effects were mechanistically linked to the modulation of systemic inflammation and the restoration of gut microbiota homeostasis. Our findings provide a strong scientific rationale for developing PA53 as a preventive strategy for at-risk individuals and underscore the critical importance of intervention timing in probiotic therapy for metabolic diseases.

## Data Availability

The datasets generated during this study are included in this article. The 16S rRNA gene sequence areavailable in the NCBI genome database repository (PRJNA1219805).

## Supplementary material

Supplementary Figure 1 **(A)** Body weight. **(B)** H&E staining of mouse livers. **(C)** H&E staining of mouse ileum. **(D, E)** Serum inflammatory factor levels and biochemical parameters. Values were expressed as mean ± SD (n = 6). CTL, control group; PA-E, *Pediococcus acidilactici* PA53 early prevention group; NAF, NAFLD group; CUR, curcumin group, PA-L; *Pediococcus acidilactici* PA53 late treatment group. Values were expressed as mean ± SD (n = 6). **p < 0.01, ****p < 0.0001 compared to the CTL group. ##p < 0.01, ###p < 0.001 compared to the NAF group.
