# Supplementary figures and images for: Efficacy of Pediococcus acidilactici PA53 in preventing high-fat diet-induced non-alcoholic fatty liver disease in mice
Source: Front Immunol. 2026 Jan 12;16:1743709. doi: 10.3389/fimmu.2025.1743709 (PMC12833037; doi:10.3389/fimmu.2025.1743709)

**A**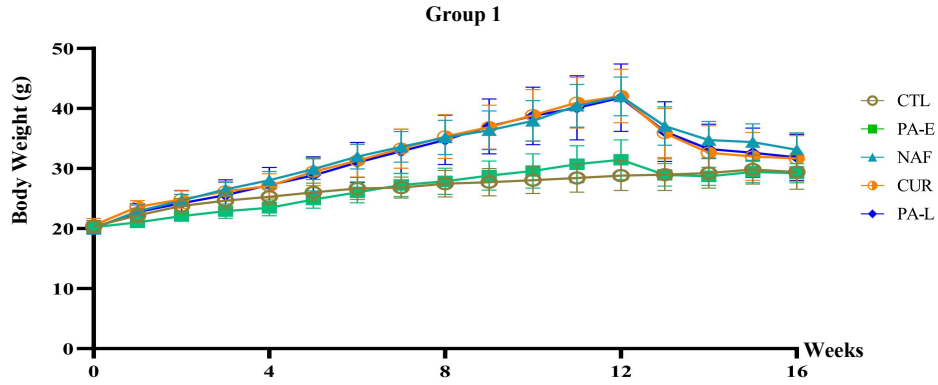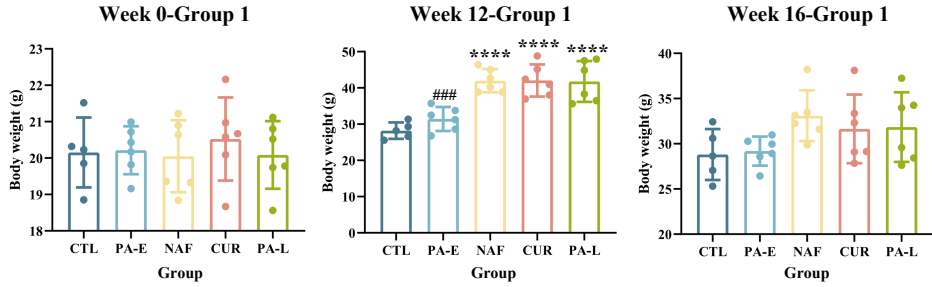**B**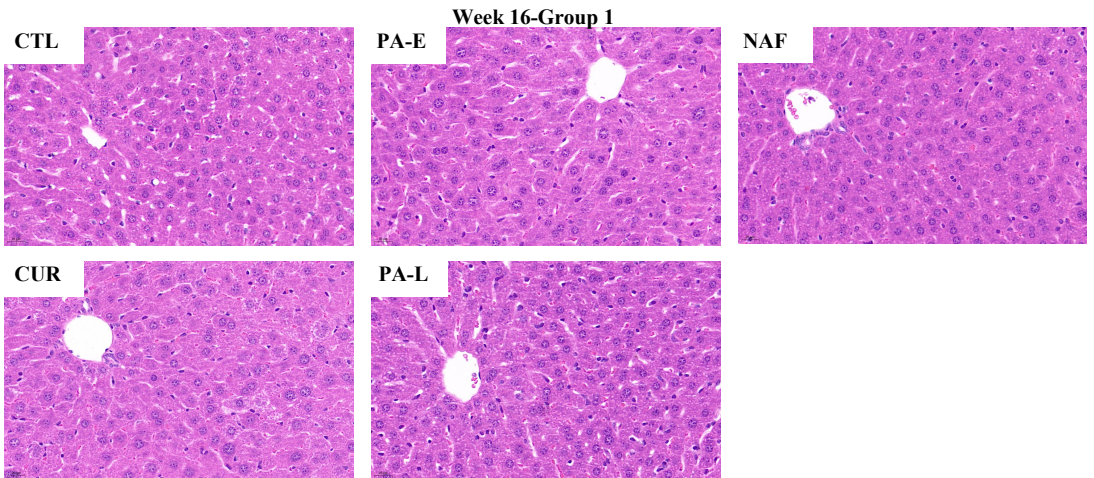

**C**

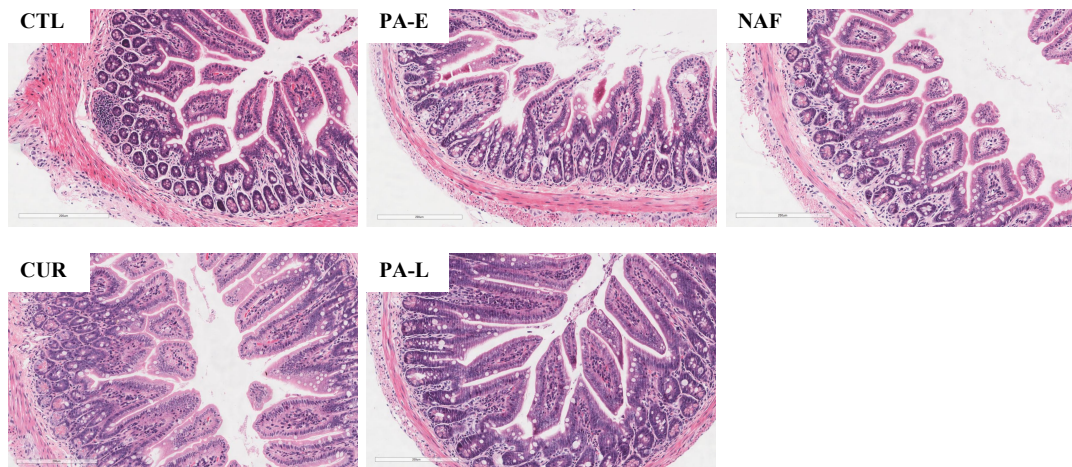

**D**

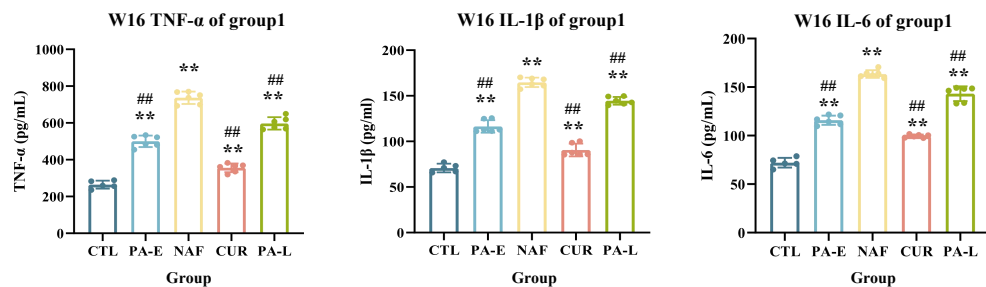

**E**

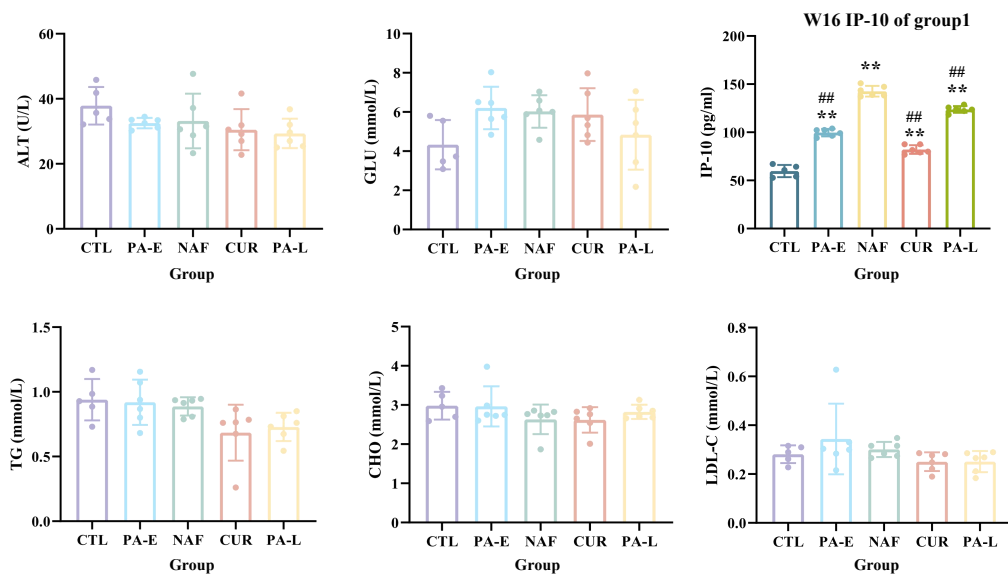

Supplement: Supplementary Figure 1 — (A) Body weight. (B) H&E staining of mouse livers. (C) H&E staining of mouse ileum. (D, E) Serum inflammatory factor levels and biochemical parameters. Values were expressed as mean ± SD (n = 6). CTL, control group; PA-E, Pediococcus acidilactici PA53 early prevention group; NAF, NAFLD group; CUR, curcumin group, PA-L; Pediococcus acidilactici PA53 late treatment group. Values were expressed as mean ± SD (n = 6). **p < 0.01, ****p < 0.0001 compared to the CTL group. ##p < 0.01, ###p < 0.001 compared to the NAF group. [file DataSheet1.pdf]
